# Epidemiology, virulence factors and management of the pneumococcus

**DOI:** 10.12688/f1000research.9283.1

**Published:** 2016-09-14

**Authors:** Charles Feldman, Ronald Anderson

**Affiliations:** 1 Charlotte Maxeke Johannesburg Academic Hospital and Faculty of Health Sciences, University of the Witwatersrand Medical School, Johannesburg, South Africa; 2 Institute for Cellular and Molecular Medicine, Department of Immunology, Faculty of Health Sciences, University of Pretoria, Pretoria, South Africa

**Keywords:** community-acquired pneumonia, adjunctive therapy, Streptococcus pneumoniae, Antibiotic resistance

## Abstract

Pneumococcal infections continue to cause significant morbidity and mortality in patients throughout the world. This microorganism remains the most common bacterial cause of community-acquired pneumonia and is associated with a considerable burden of disease and health-care costs in both developed and developing countries. Emerging antibiotic resistance has been a concern because of its potential negative impact on the outcome of patients who receive standard antibiotic therapy. However, there have been substantial changes in the epidemiology of this pathogen in recent years, not least of which has been due to the use of pneumococcal conjugate vaccines in children, with subsequent herd protection in unvaccinated adults and children. Furthermore, much recent research has led to a better understanding of the virulence factors of this pathogen and their role in the pathogenesis of severe pneumococcal disease, including the cardiac complications, as well as the potential role of adjunctive therapy in the management of severely ill cases. This review will describe recent advances in our understanding of the epidemiology, virulence factors, and management of pneumococcal community-acquired pneumonia.

## Introduction

Throughout most regions of the world, community-acquired pneumonia (CAP) has been documented to be associated with a significant clinical and economic burden of disease ^1– 5^. Much of this burden of disease is due to *Streptococcus pneumoniae* (pneumococcus), since this microorganism is regularly documented to be the most common bacterial cause of CAP in the vast majority of studies ^1– 5^. The purpose of this article, which is largely an update of a similar one published in 2014 ^6^, is to review the latest information regarding the epidemiology, virulence factors and management of pneumococcal disease, with a particular focus on pneumococcal pneumonia.

## Epidemiology of pneumococcal disease

### The burden of pneumococcal pneumonia

Pneumococcal infections are characterized as being invasive or non-invasive ^7^. Invasive pneumococcal disease includes meningitis and bacteremia, and non-invasive disease includes otitis media and sinusitis. With regard to pneumococcal pneumonia specifically, these infections may be either invasive (bacteremic) or non-invasive (non-bacteremic) ^7^. A systematic review and meta-analysis of the literature that provided data on the yield of the various diagnostic techniques available for confirming the presence of pneumococcal pneumonia clearly indicated that the true burden of disease is considerably underestimated when these assessments are based on data from bacteremic infections alone ^8^. In fact, it has been said that for every case of bacteremic pneumococcal CAP there are approximately three additional cases of non-bacteremic pneumonia. Non-bacteremic pneumonia represents the largest burden of pneumococcal infection in adults ^7^ and therefore is the focus of the present review.

### The role of *Streptococcus pneumoniae* in the etiology of community-acquired pneumonia

A number of studies from Europe, including a recent literature review, while noting that the prevalence of *S. pneumoniae* in CAP does vary in different regions and in different clinical settings, indicated that this microorganism was the most commonly isolated pathogen in patients with CAP ^9^. Furthermore, in that literature review, the frequency of isolation of *S. pneumoniae* was higher in patients who were at least 65 years old than in younger adults, and *S. pneumoniae* remained the most common isolate in patients who were HIV-infected and in those cases who had chronic obstructive pulmonary disease (COPD). In another systematic review and meta-analysis investigating the role of *S. pneumoniae* in CAP, this microorganism was more likely to be detected in studies in which polymerase chain reaction assays were performed for diagnostic purposes, and also in studies including intensive care unit (ICU) cases compared with that observed in hospital-treated patients or outpatients ^10^. Other studies from Europe have also concluded that the pneumococcus is the most commonly isolated pathogen in CAP, being a cause of 35% of cases overall ^7^. Additional studies have also reported that the pneumococcus was the most common pathogen irrespective of whether the patients were treated in the outpatient, inpatient or hospital settings, including younger patients and even cases with nursing home-acquired pneumonia ^7^. A study from the German Network for Community-acquired Pneumonia (CAPNETZ) investigating 7,400 cases from 12 clinical centers documented a pathogen in 32% of the patients with CAP, and among the latter cases the pneumococcus was the most commonly isolated pathogen (30% of patients) ^11^. Compared with patients with non-pneumococcal infections, those with pneumococcal pneumonia were more likely to be admitted to hospital, to have a higher CURB-65 score (confusion of new onset [abbreviated mental test score of 8 or less], blood urea nitrogen of greater than 7 mmol/l (19 mg/dl), respiratory rate of 30 breaths per minute or greater, systolic blood pressure of less than 90 mm Hg or diastolic blood pressure of 60 mm Hg or less, and age of at least 65 years), and to have a pleural effusion more frequently as well as a need for mechanical ventilation and oxygen therapy ^11^.

Two recent studies, which included data on microbial etiology, were reported from the US among adults hospitalized with CAP ^12, 13^. In both studies, rhinovirus was the most common isolate. In the first study, which included both invasive and non-invasive cases, the pneumococcus was the second most common isolate, representing 18.5% of cases in which an etiological agent was identified ^12^. The second study, despite an extensive microbiological investigation which included molecular diagnostic testing, documented a pathogen in only 38% of cases (853 out of 2,259 patients) ^13^. The pneumococcus was the third most common isolate, being documented in 5% of patients. However, the authors conceded that there may be a number of reasons for the low yield of pathogens despite a comprehensive diagnostic approach, including an inability to obtain adequate lower respiratory tract samples, antibiotic exposure prior to diagnostic testing, and insensitivity of diagnostic tests for known pathogens ^13^. Furthermore, not all eligible patients were enrolled, those who were at least 65 years old and/or were undergoing mechanical ventilation were less likely to be enrolled and not all enrolled patients had all diagnostic studies done. The authors also suggested that the low yield specifically of pneumococcal isolates may also relate to the indirect effects (“herd protection”) of pediatric pneumococcal vaccination. 

### Antibiotic resistance in pneumococcal community-acquired pneumonia isolates

Antibiotic resistance is known to be an important issue in pneumococcal infections ^14, 15^, and in the literature review described above, penicillin resistance was variably reported in 14.9% to 25.7% and erythromycin resistance in 12.0% to 21% of pneumococcal isolates causing CAP ^9^. Penicillin resistance in pneumococci is defined by the minimum inhibitory concentration (MIC) breakpoints that are determined by the Clinical and Laboratory Standards Institute (CLSI) and the European Committee on Antimicrobial Susceptibility Testing (EUCAST) ^16^. The classic CLSI MIC breakpoints, established to prevent likely treatment failures in pneumococcal meningitis, defined penicillin-susceptible pneumococcal isolates as those having an MIC of not more than 0.06 mg/l, intermediate resistance strains as those having an MIC of between 0.12 and 1 mg/l, and resistance strains as those having an MIC of at least 2 mg/l. These MIC levels are still considered appropriate for the evaluation of meningitis treatment and possibly oral treatment of non-meningeal pneumococcal infections. However, for parenteral treatment of pneumococcal pneumonia, the breakpoints now considered appropriate are an MIC for susceptible strains of 2 mg/l or less, for intermediate strains of 4 mg/l and for resistant strains of 8 mg/l or more ^16^.

Macrolide resistance is predominantly due to either an efflux pump mechanism, which tends to be associated with lower levels of macrolide resistance and is common in North America, or a ribosomal methylation mechanism that tends to be associated with higher levels of macrolide resistance and is more common in Europe ^16^. Pathogens with both mechanisms of resistance are also emerging.

However, there has been considerable debate as to whether current prevalence and levels of resistance have a true impact on the outcome of patients who receive standard guideline-compliant therapy, particularly with regard to the macrolide group of antibiotics ^14, 16– 19^. In pneumococcal pneumonia, as opposed to meningitis, there is considerably less concern, in general, with regard to beta-lactam resistance because of the high levels that the commonly used antibiotics can achieve in blood and tissues, and the only concern is in the case of isolates with very high MICs ^20^. These, fortunately, remain rather uncommon at the current time in both Europe ^21^ and the US ^22^, and in some studies their numbers have actually decreased in both children and adults following the introduction of the pneumococcal conjugate vaccine (PCV) in children. However, researchers have certainly voiced concern about escalating levels of macrolide resistance, citing evidence of failure of macrolide therapy in cases with both low-level and high-level macrolide resistance, and have therefore indicated that macrolide monotherapy for CAP may not be appropriate ^16, 17^. One study investigating the effects of macrolide resistance on presentation of pneumococcal pneumonia and its outcome indicated that patients with macrolide-resistant *S. pneumoniae* infections were not more severely ill compared with those with antibiotic-susceptible infections, nor did they have worse clinical outcomes ^18^. Thus, it is widely recommended that macrolides still be used routinely in CAP, generally as part of combination therapy for patients with severe CAP and sepsis (see antibiotic treatment) ^19^. It is important to reflect on the fact that implementation of the PCVs in children, which is discussed in more detail below, has had a significant impact on the occurrence of drug-resistant pneumococcal infections in both children and adults ^20, 22^.

### Invasive pneumococcal disease

It has been said that pneumococcal pneumonia is associated with bacteremia (invasive pneumococcal pneumonia, or IPD) in approximately 10% to 30% of cases ^14^. Before the widespread introduction of the PCVs in children—with subsequent “herd protection” which was associated with a decreased incidence of pneumococcal infections even in unvaccinated children and adults—the rates of incidence of IPD in Europe and the US were reported as being between 11 and 49 cases per 100,000 population ^7, 14^ and between 16.2 and 59.7 out of 100,000 in adults more than 65 years old ^14^. The incidence was considerably higher in cases with underlying co-morbid conditions (176 to 483 cases per 100,000 population) and highest in those with underlying immunosuppression (342 to 2,031 per 100,000 population) ^14^. The incidence of IPD is affected by many factors, including the use of pneumococcal vaccination in children and in adults through direct or herd protection (or both) ^7^. Considerable reductions in the rate of vaccine-type IPD have been documented in all ages following introduction of the PCVs in childhood programs, particularly in the US ^23^. Sustained declines in hospitalizations for CAP in both children and adults were documented in the US following the introduction of PCV7 ^24^, and early data have shown similar further benefits with the introduction of PCV13, not only in vaccinated children but also in at least some of the adult age groups ^25^.

However, some European studies, particularly those in France, have indicated an increase in the incidence of IPD in adults, particularly in cases with underlying risk factors, which have been ascribed to various possibilities including differences in vaccine coverage compared with that of the US, fluctuations in serotypes, and possible outbreaks of infection ^7, 26^. Certainly, following introduction of PCV7, many, but not all, studies documented the occurrence of serotype replacement disease ^27^. This was an increase in invasive pneumococcal infections due to non-vaccine serotypes, in particular 19A, but including other serotypes, occurring in both children and non-vaccinated adults ^27^. The magnitude of the replacement in non-vaccinated groups varied, and some areas reported complete serotype replacement, indicating no net change in overall IPD incidence, whereas other areas reported very little serotype replacement ^27^. ****Subsequently, PCV7 use in children was largely replaced by PCV13, the latter also covering for serotype 19A, and while a similar reduction in vaccine-type IPD was also documented, as with PCV7, serotype replacement disease is once again being documented ^28, 29^.

Whereas a study from England and Wales showed a reduction in IPD following introduction of the conjugate vaccines with substantial herd protection initially from PCV7 and extending subsequently to PCV13 ^30^, an additional study in adults from the UK indicated that although reductions in pneumococcal infections have occurred in adults (in association with herd protection), vaccine-type pneumococcal disease continues to have a high burden of disease in adults in that country ^31^.

### Risk factors for pneumococcal infections

Although a detailed description of risk factors that increase the incidence of pneumococcal infections is beyond the scope of the present review and has been extensively reviewed elsewhere ^32, 33^, some pertinent or recent studies (or both) need specific mention. It is clearly indicated that although immunosenescence, which is age-related deterioration in host immune responses in the elderly, is well recognized as a risk factor for infections in general, it is poorly understood and is often undetected ^34^. Cigarette smoking is recognized to be one of the most important independent risk factors for IPD among immunocompetent non-elderly adults ^35^, such that smoking strategies have the potential to reduce the risk of IPD significantly. In addition, recent data have suggested that current smokers with pneumococcal pneumonia have an increased risk of severe sepsis, require hospitalization at a younger age, despite fewer comorbidities, and have an increased risk of 30-day mortality independent of age and comorbidity ^36^. HIV infection, as a risk factor for pneumococcal infections, is also well characterized ^37^. Recent systematic literature reviews from Europe and Canada have confirmed the importance of various lifestyle and comorbid conditions—including smoking, alcohol abuse, being underweight, poor dental hygiene, chronic respiratory conditions such as COPD and asthma, diabetes mellitus, chronic heart disease, chronic liver disease, cerebrovascular disease, Parkinson’s disease, epilepsy, dementia, dysphagia, and chronic renal and liver disease—as risk factors for pneumococcal CAP ^11, 38– 40^.

## Pneumococcal virulence

This section covers recently described roles for established pneumococcal virulence factors—specifically the cholesterol-binding, pore-forming toxin, pneumolysin (Ply); the adhesin, choline-binding protein A (CbpA); and hydrogen peroxide (H _2_O _2_)—as well as a brief overview of novel virulence factors, some with vaccine potential, not included in our previous reviews ^6, 41, 42^. ****This is preceded by a consideration of the emerging threat posed by non-encapsulated strains of the pneumococcus coincident with the widespread inclusion of PCVs in national childhood immunization programs.

### Non-encapsulated *Streptococcus pneumoniae*

The polysaccharide capsule of the pneumococcus is widely recognized as being the major virulence determinant of this bacterial pathogen and accordingly the primary target for vaccine design. Though efficacious, capsular polysaccharide-based vaccines have two significant limitations: firstly, the restricted immunogenicity of the capsular polysaccharides, which results in direct activation of B cells without the involvement of T-cell help, which can be overcome by conjugation to a protein carrier, and, secondly, the large number of pneumococcal capsule structural variants, known as serotypes. Based on conventional serotyping procedures, complemented by molecular analyses, at least 97 different serotypes of the pneumococcus have been identified ^43^. Consequently, the number of different capsular polysaccharides contained in pneumococcal vaccines is limited to those serotypes most frequently associated with severe disease ^42^.

Two types of pneumococcal vaccines are currently licensed for use in humans and vary with respect to design, composition, target populations, immunogenicity and efficacy. These are pneumococcal polysaccharide vaccine 23 (PPV3) and several types of PCV ^42^. PPV23, licensed in the US in 1983, consists of capsular polysaccharides derived from 23 different serotypes of the pneumococcus, which collectively account for 85% to 90% of cases of IPD ^42, 44^.

The first PCV, PCV7, was licensed in the US in 2000 but has been superseded by PCV13, which consists of 13 capsular polysaccharides, derived from the most common disease-causing serotypes of the pneumococcus. Relative to PPV23, PCVs have markedly improved immunogenicity in neonates and young children. This has resulted in their widespread and successful inclusion in the national childhood immunization programs of many developed and developing countries ^24, 30, 45– 57^. Importantly, unlike PPV23, PCVs do counter nasopharyngeal carriage of vaccine strains of the pneumococcus, resulting in induction of indirect immunity, also known as herd immunity, as mentioned above, thereby conferring secondary protection on adults ^24, 30, 49, 50, 53, 56, 57^.

Despite their undoubted impact in preventing IPD across all age groups (vaccinated and non-vaccinated), PCVs do have limitations, as alluded to above. Notwithstanding an increased frequency of nasopharyngeal colonization by non-vaccine serotypes of the pneumococcus, the global implementation of PCV13 and its forerunners in national childhood immunization programs has also been accompanied by the emergence of non-encapsulated strains of the pathogen ^58^. These have been reported to account for an estimated 3% to 19% of asymptomatic carriage isolates, with the lower figure possibly representing a more realistic estimate ^58^. Though somewhat less virulent than their encapsulated counterparts, these non-encapsulated strains of the pneumococcus nevertheless have a significant association with non-invasive infections, such as conjunctivitis and otitis media, as well as with IPD, albeit at a lower prevalence ^58^.

In addition to anti-phagocytic activity, the polysaccharide capsule plays a role in nasopharyngeal colonization by enabling the pathogen to evade attachment to airway mucus, thereby interfering with expulsion of the pathogen by the mucociliary escalator ^59^. However, a reduction in capsule size is necessary to expose the various underlying protein adhesins which mediate attachment to respiratory epithelium, a prerequisite for robust colonization and invasion ^60^. Loss of the polysaccharide capsule appears to involve repression of the genes involved in capsule production which reside in a single cluster known as the *cps* locus ^58, 61^. However, mutations or deletions of *cps* genes may result in the emergence of non-encapsulated strains of the pneumococcus ^58^.

The loss of the capsule in non-encapsulated strains of the pneumococcus, however, is counter-balanced by the acquisition of a range of compensatory virulence mechanisms, some unique and others related to increased expression of existing mechanisms. Foremost in the former category is expression of the novel adhesin, pneumococcal surface protein K (PspK), encoded by the *cps* replacement gene, *pspK*, which has been described in a non-encapsulated subtype of the pneumococcus ^58, 60^. PspK promotes adhesion of the non-encapsulated pathogen to the respiratory epithelium of the host, albeit by poorly characterized mechanisms, which nonetheless appear to contribute to nasopharyngeal colonization ^58, 60, 62, 63^. In addition, PspK may contribute to the virulence of non-encapsulated pneumococci by binding to, and neutralizing, secretory IgA on mucosal surfaces ^58, 62^.

Increased production of biofilm by non-encapsulated strains of the pneumococcus, also favoring colonization or virulence (or both), is another mechanism which distinguishes these strains from their capsulated counterparts ^58, 64^. Biofilm is a self-generated, polymer matrix which insulates the pathogen against host defenses and antimicrobial agents, enabling it to remain quiescent, re-emerging when the host environment is less hostile. In this context, it is noteworthy that acquisition of the non-encapsulated phenotype in a *cspE* gene mutant of serotype 18C of the pneumococcus was found to be associated with increased expression of six early competence pathway genes involved in DNA binding, uptake and recombination, as well as of one competence-pathway-associated gene, the expression levels of these being 11- to 34-fold higher than the wild-type encapsulated variant ^65^. The *cspE* gene encodes a glycosyltransferase enzyme which catalyses the first step in capsule formation ^58, 65^. In addition to involvement in biofilm formation, increased expression of competence genes was associated with increased growth, a 117-fold increase in adhesion to nasopharyngeal epithelial cells, and enhanced genetic transformability ^65^; the last of these probably underpins the higher rates of antibiotic resistance reported in non-encapsulated strains of the pathogen ^58^.

Notwithstanding the remarkable impact of PVCs in particular, the emerging threats posed by nasopharyngeal colonization with non-vaccine serotypes and non-encapsulated strains of the pneumococcus clearly underscore the need for future generation pneumococcal vaccines designed to provide much broader protective coverage. In this context, non-encapsulated whole cell vaccines which express immunogenic proteins, adhesins, or attenuated virulence factors common to capsulated and non-encapsulated strains of the pneumococcus (or both) are currently in the developmental pipeline ^42^. Alternatives include vaccines consisting of recombinant proteins expressed by both types of the pneumococcus ^42^. These novel vaccines are likely to complement PCVs rather than replace them. In this context, however, it is noteworthy that non-encapsulated strains of the pneumococcus may not express two of the major protein vaccine candidates, PspA and PspC, expressed by encapsulated strains ^58^.

### Novel roles in tissue injury for established pneumococcal virulence factors

The association between pneumococcal pneumonia and acute cardiac events was first described by Musher *et al*. in 2007 ^66^. These authors reported that “patients with pneumococcal pneumonia are at substantial risk for a concurrent acute cardiac event such as myocardial infarction, serious arrhythmia, or new or worsening congestive heart failure” ^66^. It is only fairly recently, however, that significant insights into the pathophysiology of myocardial damage associated with IPD have emerged following the publication of data derived from two independent experimental animal studies.

In the first of these, Brown *et al*. reported on the occurrence of invasion of the myocardium during experimental pneumococcal infection of mice and rhesus macaques ^67^. Myocardial invasion was dependent on the expression of two pneumococcal adhesins, CbpA and cell wall phosphorylcholine expressed on the lipoteichoic acid backbone, which interact with laminin receptors on vascular endothelial cells and the platelet-activating factor receptor, respectively ^67– 69^. Invasion of the myocardium resulted in the development of cardiac microlesions and myocardial damage due predominantly to the cytotoxic actions of Ply. These findings were supported by observations of similar microlesions in cardiac sections from patients who had succumbed to IPD ^67^. In addition, a subsequent study by Alhamdi *et al*. confirmed the critical involvement of Ply in the pathogenesis of cardiac injury in a murine model of experimental IPD ^70^. Notable differences between the studies by Brown *et al*. ^67^ and Alhamdi *et al.* ^70^ include the apparent lack of involvement of CbpA and cardiac colonization in the latter study, in which circulating Ply appeared to be the sole mediator of myocardial damage.

Additional mechanisms by which Ply may promote cardiotoxicity in IPD include secondary cytotoxicity due to release of histones from dead and dying cardiomyocytes and other types of bystander cells ^71^. In addition, Ply has been reported to activate platelet aggregation *in vitro*, which, if operative *in vivo*, may also contribute to myocardial dysfunction via platelet plug formation and microvascular damage ^72, 73^.

In a more recent study, Gilley *et al*., while confirming the involvement of Ply in the pathogenesis of cardiac microlesions in a murine model of experimental IPD, also reported that Ply induces a type of inflammatory cell death known as necroptosis in infiltrated macrophages, possibly contributing to persistence of the pneumococcus ^74^. In addition, and in contradistinction to the earlier studies ^67, 70^, these authors reported that cardiac invasion by a Ply-deficient mutant of the pneumococcus also resulted in the formation of microlesions. Although the exact pneumococcal cytotoxins involved in this type of Ply-independent cardiotoxicity were not identified, the authors speculated that H _2_O _2_ produced via the activity of pneumococcal pyruvate oxidase may be implicated. In this context, low and high concentrations of H _2_O _2_ induce apoptosis and necroptosis, respectively, in eukaryotic cells ^75, 76^. The pneumococcus, which is a catalase-negative microorganism, appears to protect itself against both self-and host-generated extracellular H _2_O _2_ through the surface expression of the anti-oxidative, thioredoxin-fold lipoproteins Etrx1 and 2, as well as the detoxifying enzyme methionine sulfoxide reductase ( *Sp*MsrAB2) ^77^. Ply in particular and possibly also H _2_O _2_ appear to represent important targets in the prevention of cardiac sequelae in patients with IPD.

### Novel pneumococcal virulence factors

This section is a brief summary of novel pneumococcal virulence factors published since our last review of these in 2014 ^42^. They are summarized in Table 1 together with the relevant references ^78– 85^. These clearly reinforce the already impressive armamentarium of virulence factors used by the pneumococcus.

**Table 1.** Novel virulence factors of the pneumococcus.

Virulence factor	Function	Outcome	Reference
PbIB (cell wall, phage-encoded platelet-binding protein)	Pro-adhesive, also binds to galactose- containing residues on lung epithelium	Promotes nasopharyngeal colonization	78
DiiA (cell wall protein)	Pro-adhesive, binds to collagen and lactoferrin	Promotes nasopharyngeal colonization and dissemination	79
GH20C (a novel, presumably cell wall, β-hexosamidase)	Involved in nutrient acquisition by processing hexosaminide sugars from host glycans	Promotes growth and persistence	80
Bg1A3 (a cell membrane 6-phospho-β-glucosidase)	Converts phosphorylated substrates to usable, nutrient monosaccharides	Promotes survival and virulence	81
Spbhp-37 (cell wall hemoglobin- binding protein)	Iron acquisition	Growth and infectivity	82
Elongation factor Tu (Tuf), a protein with both cytoplasmic and cell surface locations	Binds inactivators of the complement systems (Factor H and complement Factor H-related protein 1) preventing complement-mediated attack	Immune evasion, virulence	83
Polyamine transporter, potABCD	Promotes uptake of polyamines which protect against acid and reactive oxygen species and promotes biofilm formation	Immune evasion, virulence	84
L-Ascorbate-6-phosphate lactonase, a protein with a cell membrane location	Highly conserved enzyme with metallo-β- lactamase activity	Possible contributor to β-lactam antibiotic resistance	85

## Antibiotic and adjunctive therapy

Several recent studies have described the optimal antibiotic management of patients with CAP, including pneumococcal CAP, in both hospitalized (including ICU cases) and non-hospitalized patients, and these have largely confirmed previous findings. One recent randomized study, largely in non-severely ill patients (mean pneumonia severity index [PSI] score of 84), indicated that in non-severely ill cases, (PSI I to III) but not in severely ill cases (PSI IV), beta-lactam monotherapy was non-inferior to a beta-lactam-macrolide combination ^86^. However, there was a non-significant trend to superiority of combination therapy, and the 30-day readmission rate was higher in the monotherapy arm ^86^. A more recent systematic literature review indicated that the lowest short-term mortality in patients with CAP was associated with the early initiation (4 to 8 hours) of a beta-lactam-macrolide combination or fluoroquinolone monotherapy ^87^. However, a further study in the ICU setting indicated that the combination therapy was associated with a lower mortality than the monotherapy and was associated with better outcomes, though not necessarily decreased mortality, in patients not requiring ICU admission but with risk factors for a poor outcome and in bacteremic pneumococcal infections ^88^. The same authors documented both in severe pneumococcal pneumonia and in severe non-pneumococcal pneumonia that early administration of antibiotics and combination antibiotic therapy was associated with improved ICU survival ^89, 90^. These studies complement the findings of a systematic review and meta-analysis of critically ill patients with CAP which documented that macrolide-containing regimens were associated with a reduced mortality compared with the use of non-macrolide-containing antibiotic regimens ^91^. A number of recent reviews have reinforced the importance of macrolide combination antibiotic therapy on the outcome of CAP, particularly among severely ill patients with CAP, highlighting the relative safety of use of these agents, even in elderly patients, while reaffirming the likely importance of the immunomodulatory effects of these agents ^92– 94^.

Since the mortality of patients with CAP remains substantial despite appropriate antibiotic use, a number of adjunctive therapies have been recommended for severely ill patients with CAP in an attempt to improve the outcome ^6, 95, 96^. Macrolide antibiotics may be considered to have both antimicrobial and adjunctive effects, the latter because of their immunomodulatory properties, which have recently been reviewed ^93, 97^. Among the other putative adjunctive therapies, corticosteroids currently appear to be the most promising agents on the basis of a number of recent studies and meta-analyses ^98– 100^. Statins and antiplatelet agents appear to be interesting because of the particular role they may play in preventing the occurrence of cardiovascular events in patients with CAP, whereas a myriad of other agents have also been mentioned, the potential benefits of which have been reviewed elsewhere ^95, 96, 101^.

## Mortality

It is interesting to reflect on the fact that despite medical advances, the case fatality rate for patients hospitalized with IPD has remained relatively constant over the past 60 years ^102^ and that the mortality of patients with pneumococcal CAP, even among patients admitted to the ICU, remains high despite appropriate antibiotic treatment ^103^. There has been much ongoing debate as to the relative impact of host factors, versus pneumococcal serotype/antibiotic therapy on the outcome of pneumococcal CAP ^104^. With regard to respiratory failure, which is said to be a frequent complication of pneumococcal pneumonia, certain serotypes are the main risk factors (serotypes 3, 19A and 19F) ^105^. However, several other studies attest to the importance of host factors, as predictors of mortality, including lifestyle factors such as alcohol use disorders ^106^, tobacco smoking ^36^, advanced age ^104, 107^, and underlying comorbidities both in hospitalized patients and in patients admitted to the ICU ^104, 108^. Interestingly, in contrast to studies mentioned above, one recent study suggested that current smoking was associated with a lower mortality in bacteremic pneumococcal pneumonia since these individuals were more likely to be infected with serotypes associated with a low case fatality rate ^109^. A number of additional studies, some described above, have indicated that there has been a declining mortality in adults with pneumococcal infections, mainly in the age group of 18 to 64 years, as a consequence of herd protection afforded by childhood vaccination with the PCVs ^110^.

## Conclusions

Despite very substantial advances in diagnosis, therapy and ICU care, the outcome of patients with IPD, particularly the elderly and those with other associated risks, has remained essentially unchanged for several decades, even in the setting of seemingly appropriate antimicrobial therapy. This has led to a heightened awareness of the existence of sub-groups of patients at particularly high risk for the development of life-threatening complications, including acute lung injury and acute cardiovascular events, who are likely to benefit most from early, aggressive antibiotic therapy, as well as discerning administration of adjunctive, anti-inflammatory therapies. Targeting high-risk sub-groups would be most efficacious if undertaken in the setting of access to rapid, reliable molecular diagnostics, together with identification of those host-derived systemic biomarkers of inflammation and tissue damage which accurately predict type of complication and outcome. With respect to prevention, dual influenza and pneumococcal immunization, particularly for the elderly, is strongly recommended.

In addition, the efficacy of current vaccines may be enhanced through the development of novel “hybrid” vaccines in which capsular polysaccharides are combined with highly conserved, immunogenic, pneumococcal proteins, shared by capsulated and non-encapsulated strains, thereby conferring much broader coverage, including protection against non-vaccine serotypes and non-encapsulated strains of the pneumococcus.

## Abbreviations

CAP, community-acquired pneumonia; CbpA, choline-binding protein A; CLSI, Clinical and Laboratory Standards Institute; COPD, chronic obstructive pulmonary disease; H _2_O _2_, hydrogen peroxide; ICU, intensive care unit; IPD, invasive pneumococcal disease; MIC, minimum inhibitory concentration; PCV, pneumococcal conjugate vaccine; Ply, pneumolysin; PPV, pneumococcal polysaccharide vaccine; PSI, pneumonia severity index; PspK, pneumococcal surface protein K.
